# Not one but two: cross-sectional associations among repeat pregnancy, maternal mental health and child cognitive outcomes among adolescent and young mothers in South Africa

**DOI:** 10.1136/bmjgh-2025-019909

**Published:** 2025-10-05

**Authors:** Lorraine Sherr, Katharina Haag, Kathryn Steventon Roberts, Lucie Cluver, Janina Jochim, Wylene Saal, Nontokozo Langwenya, Camille Wittesaele, Janke Tolmay, Marguerite Marlow, Elona Toska

**Affiliations:** 1 Institute for Global Health, University College London, London, UK; 2 Norwegian Institute of Public Health, Oslo, Norway; 3 Department of Social Policy and Intervention, University of Oxford, Oxford, UK; 4 Department of Psychiatry and Mental Health, University of Cape Town, Cape Town, South Africa; 5 Sol Plaatje University, Kimberley, South Africa; 6 London School of Hygiene and Tropical Medicine, London, UK; 7 Centre for Social Science Research, University of Cape Town Faculty of Humanities, Cape Town, South Africa; 8 Institute for Life Course Health Research, Stellenbosch University, Stellenbosch, South Africa; 9 Department of Sociology, University of Cape Town, Cape Town, South Africa

**Keywords:** Maternal health, Child health

## Abstract

**Background:**

Globally, adolescent mothers and their children have poorer health outcomes. However, little is known regarding having multiple children as an adolescent. Analyses explore associations between having multiple versus single children on young mothers’ mental health and having a sibling and child development outcomes for their children. Furthermore, maternal age when having a second child (eg, multipara adolescent or multipara adolescent–adult pregnancy) is examined in relation to maternal and child outcomes.

**Methods:**

Data are drawn from a cohort of young mothers (n=1017; 10–24 years) and their children (0–68 months) residing in the Eastern Cape Province, South Africa. Effects of having multiple versus single children on maternal mental health and child cognitive outcomes (assessed using the Mullen Scales of Early Learning) were explored using hierarchical regression models. We examined associations among primipara adolescent motherhood, multipara adolescent motherhood and multipara adolescent–adult motherhood, and child cognitive development scores.

**Results:**

Poor maternal mental health was elevated among multipara mothers. Multipara mothers were more likely to report higher parental stress scores and lower social support scores (p=0.002–0.038). Child cognitive development scores were higher in children born to multipara mothers (those with a sibling *(B=*6.75, 95% CI 1.00 to 12.51, p=0.021)); younger child age (*B=*−0.56, 95% CI −0.68 to –0.44, p=<0.001) and formal childcare attendance (*B=*3.58, 95% CI 0.03 to 7.13, p=0.048) were also identified as positive predictors of higher cognitive development scores. First-born children of multipara adolescent mothers appeared to perform equally well to children born to primipara mothers (children without siblings), while first-born children of multipara adolescent–adult mothers seemed to benefit strongly from having siblings (*B*=14.31, 95% CI 4.18 to 24.44, p=0.006).

**Conclusions:**

Having multiple children was associated with poorer maternal mental health. Delaying a second pregnancy until adulthood may have benefits, as sibling effects were found to be positively associated with child cognitive development scores. Formal childcare support was associated with positive child outcomes. Findings highlight the need to focus on repeat adolescent pregnancy, improve social, psychological and family planning support among young mothers with a focus on birth timing and spacing, early childhood care provision and support for young families.

**WHAT IS ALREADY KNOWN ON THIS TOPIC**

- Adolescent pregnancy and motherhood have been found to be associated with poorer health, well-being and social outcomes for both mother and child, yet there is a dearth of literature focusing on the experience and implications of repeated pregnancy during adolescence.

**WHAT THIS STUDY ADDS**

- This is the first exploratory study globally to explore maternal well-being and child cognitive development within a sample experiencing multipara adolescent pregnancy within the context of HIV.
- Having multiple children as an adolescent/young mother was linked to poorer maternal mental health, and timing of second pregnancy (ie, delaying until adulthood) may hold more positive effects for children.

**HOW THIS STUDY MIGHT AFFECT RESEARCH, PRACTICE OR POLICY**

- There remains a need for further research to explore the experience of repeat adolescent pregnancy.
- Family planning support and early childcare provision interventions may be of benefit to this population; however, adaptation may be required to meet the needs of adolescent parent families.

## Introduction

 Ensuring maternal health and the prosperous development of children remains priorities of the sustainable development goals and, thus, remains at the core of global health and development agendas. Yet, for support interventions to be effective and economically viable, identifying potential risk among core population groups is critical. Adolescent motherhood (10–19 years)^1^ remains a salient global health issue.^2^ Globally, approximately, 10% of births are to adolescents.^2^ Within sub-Saharan Africa, such rates are elevated (pregnancy prevalence is ~22% (95% CI 19% to 26%) among 10–19 year olds and 24% (95% CI 18% to 30%) among females aged 24 years and under).^3^ While investigations into the needs of adolescent/young mothers and their children within such contexts have started (eg, Toska *et al*^4^ and Steventon Roberts *et al*^5^), existing studies predominately focus on single pregnancies and/or first-born children. There remains a dearth of literature exploring multiple pregnancies in this age group and especially within the context of other comorbid experiences, such as living with HIV. This study provides an initial exploration of the association between multiple pregnancies and maternal well-being, as well as the relationship between having a sibling and child cognitive development scores among children born to young mothers in South Africa.

Sibling families are the most prevalent family form worldwide, particularly within sub-Saharan Africa.^6^ Siblings can provide a unique context for learning and development.^7^ Historically, it has often been assumed that older siblings unidirectionally influence the development of younger siblings due to their further advanced social, cognitive, emotional and motor skills (eg, Tucker *et al*^8^). However, recent studies suggest that next to such hierarchical relationships, reciprocal influences between siblings start to emerge from the second year of the younger sibling’s life.^9 10^ As such, siblings have been found to bidirectionally influence each other’s health and activity levels, motor development, empathy levels and internalising and externalising problems.^11 12^ In addition, maternal health and well-being have previously been found to impact child development outcomes, with poor maternal outcomes being associated with worse child development.^13^,^17^ However, again, this relationship may be bidirectional with child development, also impacting on maternal health and well-being outcomes. The existing literature relating to the impacts of having multiple children (as multiples within a single pregnancy or sequentially) on maternal well-being is somewhat mixed.^18 19^ Further research is required to untangle the existing research findings, particularly within core populations, such as those mothers impacted by chronic health issues and those living with HIV or adolescent mothers.

There are many factors that influence child outcomes, some of which coexist in clusters with ramifications seldom disentangled. For example, South Africa has the largest HIV epidemic in the world, with approximately a fifth of the population (15+years) living with HIV.^20 21^ Familial HIV infection is associated with a myriad of emotional and financial stressors, including parental death, stigma and poverty.^22 23^ Direct and secondary effects (eg, depression) of the illness may also negatively affect parental caregiving.^24^ The clustering of HIV within communities means that negative effects may extend beyond the individual family and to children’s broader environment. There is limited evidence on how having a sibling would affect child development in an HIV context. Generally, evidence shows that, at an older age, siblings can pose valuable sources of support for each other in HIV-affected families.^25^ At a younger age, multiple outcomes are conceivable.^26^ On the one hand, a sibling may pose a source of cognitive stimulation, emotional support^27^ and social interaction that could support adaptive child development. On the other hand, having a sibling may thinly stretch already limited family resources, enhance conflict^28^ and divert maternal resources with a mother not only having to divide economic resources but also her attention between several children, who may have differing needs.^29^ Further research is needed on how having multiple children affects child outcomes in this context. This is especially pertinent when the sibling is born due to repeat adolescent pregnancy.

Adolescent pregnancy and birth spacing are both areas of note^30 31^ within the exploration of repeat motherhood.^32^ Maternal age (particularly at the younger end of the spectrum (ie, adolescent pregnancy)) has previously been found to impact on child development trajectories.^33^,^35^ Such impacts may operate through a complex interplay of maternal characteristics and environmental stressors (eg, lower educational attainment, financial instability and reduced emotion regulation) highlighting the multifaceted ways in which adolescent pregnancy may impact on child outcomes.^33^ Both adolescent pregnancy^36^ and maternal HIV have been found to negatively affect child development outcomes (eg, Steventon Roberts *et al*^35 37^). Yet, it remains unclear how having a sibling would affect development in children growing up with these intersecting risks. Furthermore, there is an increasing body of literature that suggests that birth spacing can affect child outcomes (eg, Rana *et al*^38^).

The current study focuses on maternal and child outcomes. To explicitly explore these issues, the current study investigated developmental outcomes of children of mothers who had a single adolescent pregnancy, a repeat adolescent pregnancy or a pregnancy during adolescence and a pregnancy in adulthood (>19 years of age; termed ‘repeat adolescent–adult pregnancy’).

## Methods

### Sample and procedure

Data are drawn from a cohort of young mothers (10–24 years) and their children, residing in the Eastern Cape province, South Africa (n=1046). Between March 2018 and July 2019, young mothers with at least one living child were interviewed. Six parallel sampling strategies were used to recruit mothers from both rural and periurban health districts (secondary schools (43 randomly selected), known health facilities (73), maternity obstetric units (9), community referrals from social workers, adolescent mothers and neighbours of adolescent mothers). All questionnaires were piloted with adolescent mothers and adolescents living with HIV (given the high prevalence of HIV within the study setting; n=34).^4^

Data gathered included: (1) detailed self-report data, focusing on sociodemographic characteristics, health, pregnancy and parenting experiences; (2) caregiver report of child data and (3) standardised child development measures administered to the child (Mullen Scales of Early Learning (MSEL)).^39^ All data collection was conducted by trained interviewers. Questionnaires were administered using electronic tablets. All questionnaires and study documents were completed in the participant’s language of choice (isiXhosa/English) and were translated. Mother–child dyads were excluded if the children were above 68 months of age (in line with the valid age range for the MSEL; n=163).^39^ Dyads were also excluded from the second step of analyses if the first child was born when the mother was >19 years of age (n=40).

## Measures

### Sibling categories

**Figure 1:**
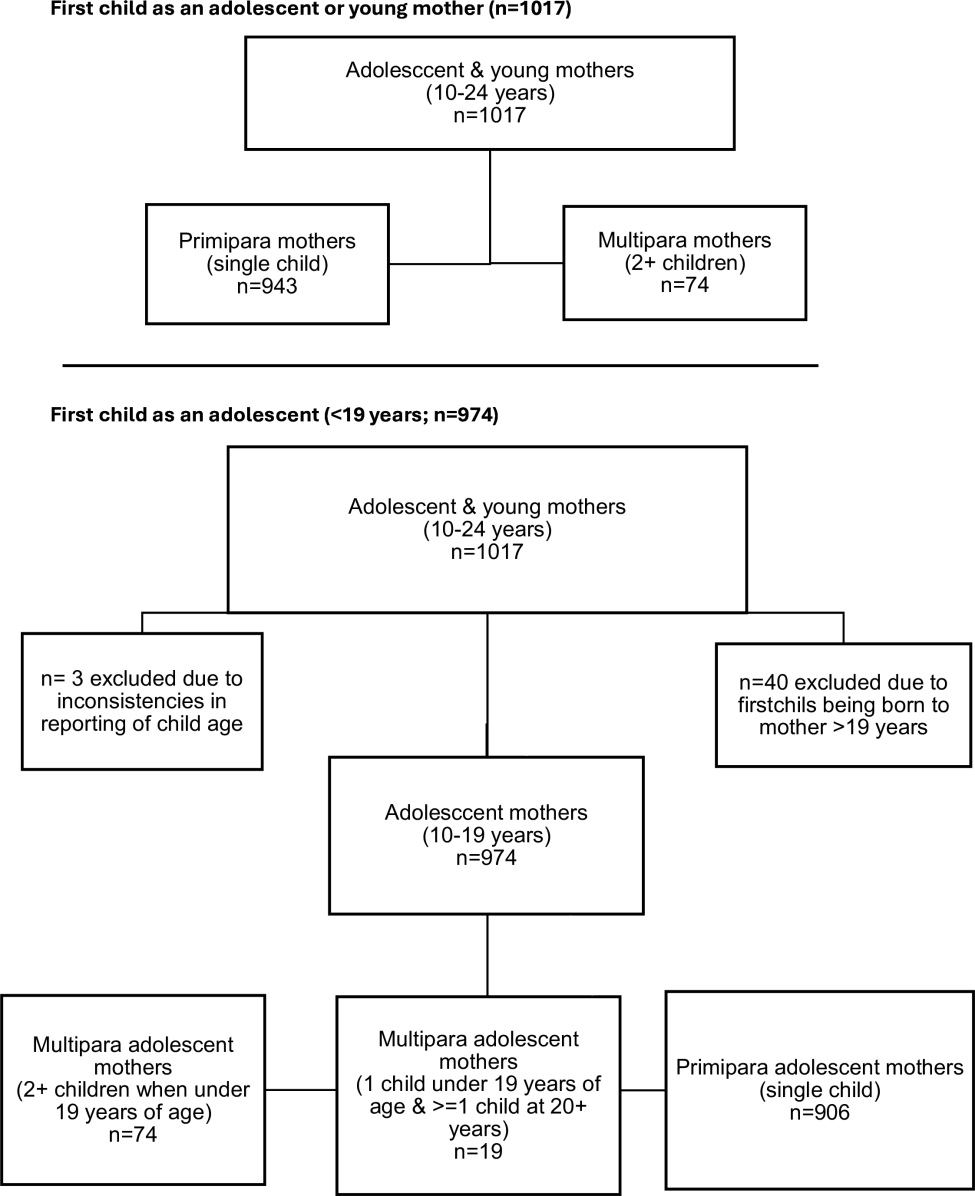
Flowchart detailing sibling categories used in the analysis.

For the first part of analyses, we investigated sibling effects on maternal and child outcomes by comparing two groups: young mothers with only a single child (primipara mothers; n=943, 92.7%) and young mothers with two or more children (multipara mothers; n=74, 7.3%). We then conducted more detailed analyses on the effects of timing and spacing of adolescent pregnancy (10–19 years), focusing on three groups: primipara adolescent mothers (1 child<=19 years; n=906, 93.0%), multipara adolescent mothers (>=2 children<=19 years; n=49, 5.0%) and multipara adolescent–adult mothers (1 child at<=19 years and>=1 child at 20+years; n=19, 2.0%). These analyses were confined to mothers with a first child born during adolescence (n=40 were excluded from full sample due to their first child being born while the mother was >19 years). Additional mothers were excluded due to inconsistencies in child age reporting (n=3 mothers indicated that their second child was older than their first child). See figure 1. All siblings were alive and were coresiding with each other.

### Maternal characteristics

Mothers provided information on their current age, age at the respective pregnancies, socioeconomic status (number of eight socially perceived necessities for children available),^40^ HIV status and if they were the child’s main caregiver. Mental health was measured by self-report on a series of validated inventories. They rated their symptoms of anxiety (Revised Children’s Manifest Anxiety Scale),^41 42^ depression (Child Depression Inventory-Short Form)^43^ and suicidality (Mini International Psychiatric Interview for Children and Adolescents—Suicidality and Self-Harm Subscale),^44^ as well as the social support they received (adjusted version of the Medical Outcomes Study Social Support Survey),^45^ and their extent of parental stress (Parental Stress Scale).^46^

### Child characteristics and child cognitive development scores

Mothers provided information on their children’s’ current age, biological sex and formal childcare attendance (in this context, this commonly comprises supervision but not necessarily activities intended to stimulate child development). Furthermore, children were administered the MSEL.^39^ Designed for use in ages 0–68 months, the MSEL captures child development across five domains: gross motor (ages<39 months only), fine motor, visual reception, receptive and expressive language (score ranges for all subscales: 20–80). A composite score (range 49–155) was calculated for the purpose of analyses. This composite score included four of the five domains of development: fine motor, visual reception, receptive language and expressive language (based on standardised scoring), and is deemed to give a composite overview of child cognitive development.^39^ While originally developed in the USA, the MSEL has also been validated for use in sub-Saharan Africa.^47^

### Statistical analyses

All analyses were conducted in Stata SE17.0. Five steps were undertaken in data analyses. First, descriptive characteristics of the sample, both overall and split by primipara and multipara pregnancy status, were obtained using χ^2^ and t-tests, as appropriate. Second, univariate hierarchical linear regression models were conducted, predicting child composite MSEL scores for children with siblings (born to multipara mothers) versus no siblings (born to primipara mothers; here termed ‘sibling effect’). Third, a range of demographic and psychosocial predictors was then added to the multivariable models if they were identified as relevant within the current literature or within univariate analyses. Due to the sample distribution, the number of possible predictors was limited within analyses. Fourth, a second set of hierarchical regression models was undertaken to investigate the effects of multipara adolescent pregnancy. For this, pregnancy status (multipara adolescent pregnancy and multipara adolescent–adult pregnancy vs a baseline group of primipara adolescent pregnancy) was included as a predictor in the univariable model (model 1) before the same demographic and psychosocial predictors as in the previous regression model were added in the multivariable model (model 2). Fifth, multivariate marginal effects models were calculated to identify mean predictive values on the MSEL to compare child development scores among children born to three groups (primipara adolescent mothers, multipara adolescent mothers and multipara adolescent–adult mothers).

## Results

### Sociodemographic characteristics

The mean age of mothers was 18.2 years (SD=1.8). Their first-born children had a mean age of 18.8 months old, with 47.3% (n=491) being female. Notably, 28.7% of mothers were living with HIV. Most mothers (92.7%, n=943/1017) had a single child (primipara), while 7.3% (n=74/1017) had two or more children (multipara). Within the multipara group, 4.8% (n=49/1017) were double-adolescent mothers (both children born before the mother turned 20), and 2.5% (n=25/1017) had their second child after reaching adulthood (age 20 or older).

Compared with only children, first-born children with siblings were older on average (t=12.25, p<0.001), more likely to attend formal childcare (χ^2^=14.11, p<0.001), and had marginally lower MSEL composite scores (t=1.84, p=0.066). Second siblings were typically 14.4 months old (SD=14.3), 57.8% female, and 16.3% attended formal childcare. Their mean MSEL scores were 94.81 (SD=21.4).

Multipara mothers were generally older (t=7.58, p<0.001) and nearly two times as likely to have HIV (χ^2^=25.04, p<0.001). They were also more likely to have repeated a school grade (χ^2^=8.16, p=0.013) and reported a higher rate of depressive (t=3.09, p=0.002), anxiety (t=3.04, p=0.002) and suicidal symptoms (t=2.59, p=0.010). Consequently, multipara mothers were more likely to screen positive on at least one mental health measure (χ^2^=5.22, p=0.038). Additionally, they reported higher parental stress (p=0.01) and less support (p=0.03), though caregiver roles did not differ between groups (p=0.25). The mother was the primary caregiver for 83.5% (n=867) of first-born children, followed by maternal grandmothers (8.2%, n=83). Very few mothers had planned their first (3.9%, n=35) or second (1.4%, n=1) pregnancies (see table 1).

**Table 1:** Sample characteristics according to primipara and multipara pregnancy status among adolescent and young women

	Overall	Primipara pregnancy (n=943)	Multipara pregnancy (n=74)	*P*
Maternal characteristics				
Age (Years; M, SD)	18.25 (1.84)	18.13 (1.79)	19.77 (1.72)	**<**0.001
Main caregiver of child(ren)	867 (85.3%)	802 (85.1%)	65 (89.0%)	0.25
Living with HIV	292 (28.7%)	252 (26.7%)	40 (54.1%)	**<**0.001
Access to basic necessities (0–8)	5.24 (2.22)	5.26 (2.23)	4.97 (2.16)	0.29
Depressive symptoms (CDI; 0–10)	0.71 (1.42)	0.68 (1.36)	1.20 (1.94)	0.002
Anxiety symptoms (RCMAS; 0–14)	0.75 (1.89)	0.70 (1.79)	1.39 (2.78)	0.003
Suicidality symptoms (MINI-Kid; 0–5)	0.18 (0.79)	0.16 (0.74)	0.41 (1.25)	0.01
Any poor mental health	128 (12.6%)	113 (12.0%)	15 (20.3%)	0.038
Parental stress score	24.79 (5.75)	24.69 (5.76)	26.17 (5.42)	0.038
Maternal social support (0–14)	13.39 (2.04)	13.43 (1.97)	12.83 (2.79)	0.018
Child characteristics				
First-born child				
Age (months; M, SD)	18.78 (14.84)	17.26 (13.76)	38.19 (14.51)	**<**0.001
Biological sex (female)	491 (47.3%)	453 (48.0%)	38 (52.1%)	0.51
Any formal childcare	233 (24.7%)	204 (23.3%)	29 (43.9%)	**<**0.001
MSEL composite score (M, SD)	93.21 (21.41)	93.55 (21.19)	88.80 (23.86)	0.066
Planned pregnancy	35 (3.4%)	33 (3.5%)	2 (2.7%)	0.73
Second-born child				
Age (months; M, SD)			14.35 (14.28)	
Biological sex (female)	–	–	42 (57.8%)	
Any formal childcare	–	–	11 (16.3%)	
MSEL composite score (M, SD)	–	–	94.81 (21.41)	
Planned pregnancy			1 (1.7%)	

CDI, Child Depression Inventory - Short Form; MSEL, Mullen Scales of Early Learning; RCMAS, Revised Children’s Manifest Anxiety Scale.

### Sibling effects: what is the relationship between having a sibling and child cognitive development scores?

Within multivariable models, a child having a sibling was linked to better composite MSEL scores (*B*=6.75, 95% CI 1.00 to 12.51, p=0.021). Older child age was negatively related to composite MSEL scores (*B*=−0.56, 95% CI −0.68 to –0.44, p=<0.0001), while formal childcare attendance was associated with higher MSEL scores (*B*=3.58, 95% CI 0.03 to 7.13, p=0.048). Overall, the model explained 10.4% in first-born children’s MSEL scores (*F*=11.81, p<0.001). See table 2.

**Table 2:** Linear regression model predicting first-born child’s developmental scores on the MSEL (N=936) based on sibling status and demographic variables

	Composite MSEL score		
Model 1	*B*	*95%* CI	*P*
Siblings (yes; multipara mother(n**=74**))	−4.76	−9.82 to 0.31	0.066
*F, p*	3.39, 0.066		
*Adjusted R^2^*	0.002		
Model 2	*B*	*95%* CI	*P*
Siblings (yes; multipara mother(n**=74**))	6.75	1.00 to 12.51	**0.021**
Maternal HIV status (living with HIV)	−0.02	−3.36 to 0.87	0.99
Maternal age (years)	0.18	−0.74 to 1.09	0.79
Access to basic necessities (0–8)	0.26	−0.34 to 0.87	0.39
Maternal depressive symptoms score (0–10)	−0.38	−1.32 to 0.56	0.43
Maternal social support score (0–14)	0.13	−0.52 to 0.77	0.70
Parenting stress score	−0.08	−0.31 to 0.14	0.47
Child biological sex (female)	−0.78	−0.40 to 1.85	0.56
Child age (years)	−0.56	−0.68 to −0.44	**<0.001**
Any formal childcare (1=yes)	3.58	0.03 to 7.13	**0.048**
*F, p*	11.81, <0.001		
*Adjusted R^2^*	0.104		

Note. Model 1: univariable analyses | Model 2: multivariable analyses | When exploring unadjusted estimates, having a sibling appears to be marginally negatively related to composite MSEL scores in the first child (*B*=−4.76, 95% CI −9.82 to 0.31), explaining 0.2% of their variance (see table 2). When including a range of relevant demographic factors in step 2 of the model, this effect was reversed through the inclusion of child age, with having a sibling now being linked to better composite MSEL scores (*B*=6.75, 95% CI 1.00 to 12.51).

Bold value signify >.05

MSEL, Mullen Scales of Early Learning.

### Repeat adolescent pregnancy effects: does having two children <20 years of age make a difference?

In basic one-way ANOVA analyses, mean cognitive development scores were found to differ significantly between groups (*F*=2.91, p=0.03), with children of primipara adolescent mothers scoring *M*=93.72 (*SD*=21.10), those children of multipara adolescent–adult mothers scoring *M*=94.84 (*SD*=26.0) and children of multipara adolescent mothers scoring lowest at *M*=84.69 (*SD*=21.78).

In step 1 of our hierarchical regression model, including only maternal status as a predictor (see table 3), multipara adolescent–adult motherhood was not associated with significant changes in child MSEL scores as compared with being primipara adolescent mothers (*B*=1.22, 95% CI −8.49 to 10.94, p=0.08), while being a multipara adolescent mother was associated with worse scores (*B*=−8.92, 95% CI −15.07 to −2.76, p=<0.004). Once controlling for age in step 2, predicted MSEL scores in both multipara groups improved. Multipara adolescent mothers’ children no longer significantly differed from the group without siblings (primipara; *B*=0.30, 95% CI −6.26 to 6.85, p=0.93), while multipara adolescent–adult pregnancy was associated with substantially higher MSEL scores (*B*=14.31, 95% CI 4.18 to 24.44, p=0.006). Again, older child age was linked to worse MSEL scores (*B*=−0.55, 95% CI −0.67 to –0.43, p=<0.001) and formal childcare attendance marginally to higher MSEL scores (*B*=3.49, 95% CI −0.08 to 7.06, p=0.056) among children. The overall model explained 10.4% of variance in child cognitive developmental scores (*F*=10.49, p<0.001).

**Table 3:** Linear regression model predicting first-born child’s cognitive developmental scores on the MSEL (N=902) based on adolescent mother status (primipara, multipara adolescent and multipara adolescent–adult) and demographic variables

	Composite MSEL score		
Model 1	*B*	*95%* CI	*P*
Primipara adolescent mother (no siblings; n=906)	Ref	–	–
Multipara adolescent–adult mother (child 1<20 years and child 2>=20 years; n=19)	1.22	−8.49 to 10.94	0.80
Multipara adolescent mother (child 1<20 years and child 2<20 years; n=49)	−8.92	−15.07 to −2.76	**0.004**
*F, p*	4.22, 0.017		
*Adjusted R^2^*	0.006		
Model 2	*B*	*95%* CI	*P*
Primipara adolescent mother (no siblings; n=906)	Ref	–	–
Multipara adolescent–adult mother (child 1<20 years and child 2>=20 years; n=19)	14.31	4.18 to 24.44	**0.006**
Multipara adolescent mother (child 1<20 years and child 2<20 years; n=49)	0.30	−6.26 to 6.85	0.93
Maternal HIV status (living with HIV)	0.09	−3.30 to 3.48	0.96
Maternal age (years)	0.27	−0.73 to 1.27	0.59
Access to basic necessities (0–8)	0.16	−0.46 to 0.77	0.62
Maternal depressive symptoms score (0–10)	−0.53	−1.48 to 0.42	0.27
Maternal social support score (0–14)	0.12	−0.55 to 0.78	0.73
Parenting stress score	−0.05	−0.28 to 0.19	0.70
Child biological sex (female)	−1.18	−3.85 to 1.48	0.38
Child age (years)	−0.55	−0.67 to −0.43	**<**0.001
Formal childcare attendance (yes)	3.49	−0.08 to 7.06	0.056
*F, p*	10.49, <0.001		
*Adjusted R^2^*	0.104		

Note. Model 1: univariable analyses | Model 2: multivariable analyses.

MSEL, Mullen Scales of Early Learning.

**Figure 2:**
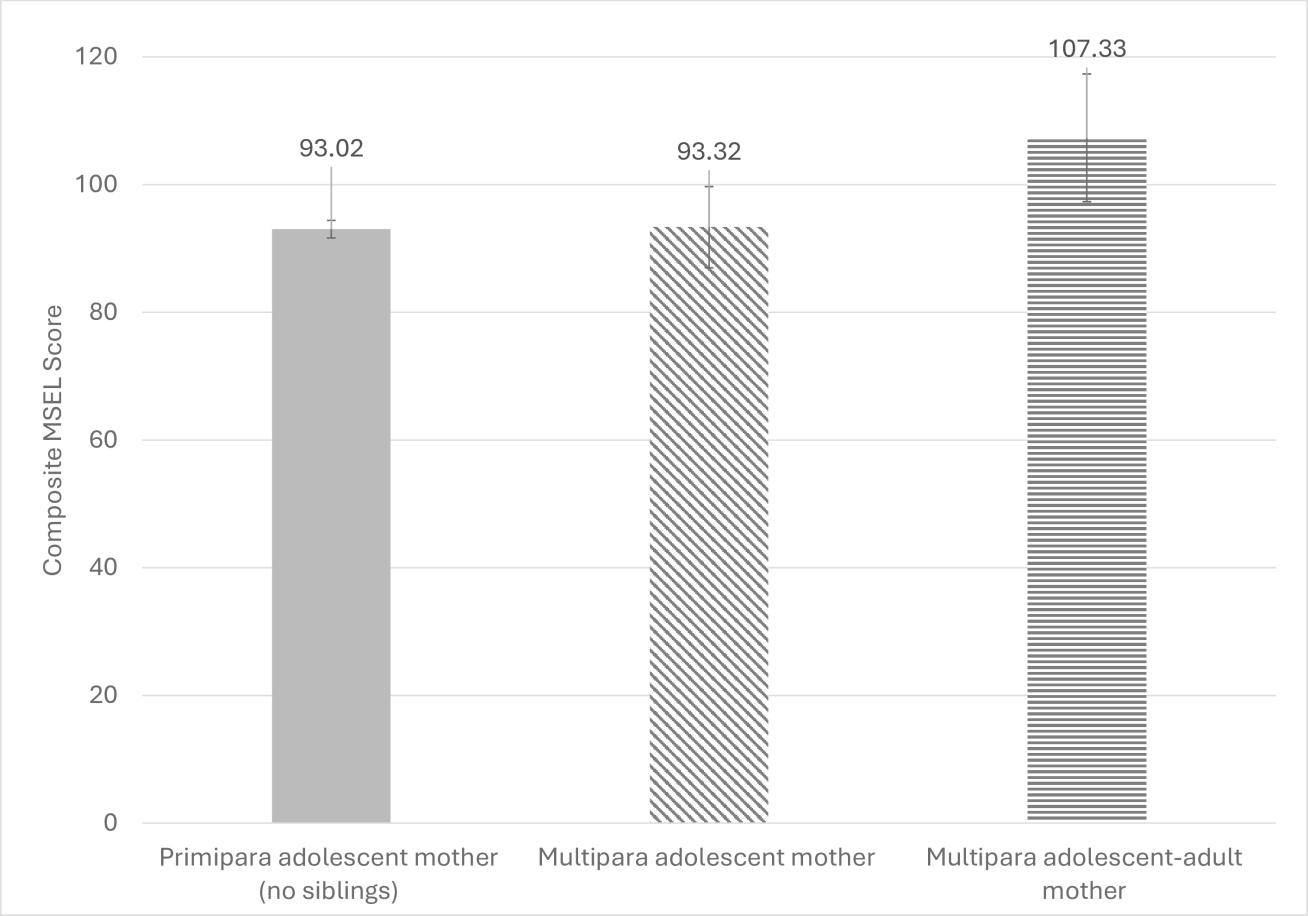
Predicted values on the MSEL (mean, 95% CI) according to maternal status, with covariates held at a constant. Note: covariates include child biological sex (female), child age (years), formal childcare attendance (yes), maternal HIV status (living with HIV), maternal age (years), access to basic necessities (0–8), maternal depressive symptoms score, maternal social support score and parenting stress score. MSEL, Mullen Scales of Early Learning.

To further explore MSEL child cognitive development scores among groups, marginal effects models were calculated. After adjusting for covariates, predicted mean values on the MSEL were M=93.02 among children born to primipara adolescent mothers, M=93.3 among children born to multipara adolescent mothers and M=107.3 among children born to multipara adolescent–adult mothers (see figure 2).

## Discussion

This is the first study globally to explore maternal well-being and child cognitive development within a sample experiencing multipara adolescent pregnancy within the context of HIV. Overall, young mothers with more than one child were more likely to experience poor mental health symptomology, greater parental stress and reported lower perceived social support compared with young mothers with more than one child. Among children, having a sibling was linked to higher child cognitive development scores. When exploring child cognitive development scores among children born to primipara adolescent mothers, multipara adolescent mothers and multipara adolescent–adult mothers, multipara adolescent–adult motherhood was associated with higher MSEL scores among children. Analyses also identified older child age as being negatively related to child cognitive development scores, and formal childcare attendance as being associated with higher cognitive development scores among children born to multipara mothers. Multipara mothers were also more likely to be living with HIV. Findings from this study contribute to the evidence base regarding adolescent and young mothers and expand on existing studies to explore the effects of giving birth to multiple children on both adolescent mothers and their children. The picture is somewhat complex, with an array of effects for both mothers and children of repeat pregnancies.

### Maternal well-being among adolescent and young mothers

Within the sample, multipara mothers were more likely to report mental health burdens, including higher depressive, anxiety and suicidality symptomology scores, and parental stress scores. Yet, despite this elevated mental health burden, multipara mothers had lower perceived social support. The direction of this effect is unclear, but our data suggest that, while support needs increase with repeat pregnancies, support provision lags. There has been a growth of interest in the mental health and well-being of adolescent and young mothers within the sub-Saharan African context.^5 37 48^ Previous literature has identified elevated poor mental health among adolescent mothers compared with adolescents who have not experienced pregnancy.^37^ There remains an absence of evidence relating to the mental health and well-being of multipara adolescent and young mothers. This initial exploration addresses this critical evidence gap, highlighting that young mothers with more than one child may be at a greater risk of poor mental health compared with young mothers with a single child. Seemingly, there is a need for mental health support among this group; however, such needs are unlikely to be met using the current mental health provision within South Africa.^49 50^ Successful existing interventions for adult mothers previously identified may require adaptation to the specific needs of young mothers, especially those living with HIV.^51^ Mental health screening and the integration of screening within existing services, such as maternal and child health services, HIV care and childcare, may also be of benefit.

### Cognitive performance among children born to adolescent and young mothers

Child cognitive development scores are seemingly impacted within the context of having more than one child as a young mother. However, this relationship is ostensibly complex. Based on the existing investigations, child cognitive development scores within the sample are lower compared with normative data from the USA and existing sample drawn from sub-Saharan Africa^39 52^—identifying this population as at risk of cognitive delay. Overall, in the sample, having a sibling (being born to a multipara mother) was linked to improved children’s development scores. However, the risk of cognitive delay is enhanced among those born to mothers experiencing repeat adolescent pregnancy, but it appears not to be driven by a sibling effect alone as, when siblings are from adolescent–adult mothers, the differences dissipate. These data would suggest that birth spacing and timing require attention among this population, and that repeat adolescent pregnancy places children at elevated risk for developmental challenges—possibly due to the compounding challenges of repeated pregnancies during a period of ongoing physical, emotional and social development.^2 4 5^ We did not examine maternal and child well-being outcomes according to whether the pregnancy was planned or unplanned, but to ensure full choice and support access to education, childcare and reproductive health clinics is a priority for adolescent mothers. However, most pregnancies were unplanned in this sample, which may have implications in relation to support and, in turn, broader impacts for maternal and child well-being. Access to childcare and reproductive health services may also allow for re-engagement with education among adolescent mothers,^53^ which in turn is likely to lead to improved outcomes for both mothers and their children. Formal childcare attendance was found to be associated with higher child cognitive development score among children, suggesting that early childhood stimulation may be a catalyst for improved child development. Given this, cognitive stimulation interventions (eg, book sharing, which has been shown to be effective in sub-Saharan Africa)^54^ may be of benefit to this population. However, such interventions may require modification to be of full benefit to adolescent mothers and their children.

### Multipara adolescent pregnancy

Although much of the adolescent pregnancy literature focuses on the prevention of adolescent pregnancy, improving access to sexual and reproductive health, particularly access to family planning, may well be needed for adolescent mothers to self-determine the timing of additional pregnancies.^31^ Adolescent mothers are more likely to report lower rates of consistent condom use and higher rates of unmet contraception need.^55^ Although siblings are common and siblings affect child development within families, having two children while still an adolescent brings with it very specific challenges, not seen if the second child is born when the mother reaches adulthood. Multipara adolescent–adult motherhood was linked with higher child cognitive development scores among their children, whereas multipara adolescent motherhood was not associated with significant variance in this sample. Repeat adolescent motherhood also came with a mental health and stress burden, yet support from family, partner or community did not necessarily follow.

### Limitations

Several study limitations should be considered within the interpretation of results from these analyses. First, the data are cross sectional. As such, causality and the direction of any associations identified within analyses cannot be wholly established. Second, the MSEL was developed in and use a reference group obtained from the USA.^39^ However, it should be noted that the MSEL has been used extensively throughout sub-Saharan Africa, including South Africa.^47^ Additionally, the independent assessment of child cognitive development is preferable compared with caregiver report. Third, while maternal HIV was adjusted for within multivariate analyses, it was beyond the scope of this study to explicitly explore child cognitive development scores among children who were living with HIV. Fourth, possible confounding factors, such as household structure and other parental characteristics (ie, maternal cognitive profile or timing of HIV diagnosis), have not been explored in these analyses. Fifth, only a small proportion of variance is explained by these models. However, child cognitive development is a complex phenomenon, and thus, even with a small variance, these analyses provide a foundational knowledge relating to specific associations that warrant further investigation. Finally, analyses do not explore findings relating to child cognitive development or maternal well-being compared with adult mothers within this setting. Such limitations restrict the generalisability of findings beyond sub-Saharan Africa. Such explorations may give further insight into the needs of young mothers and their children within this context.

## Conclusions

These findings address a critical evidence gap concerning repeat motherhood among adolescents and young women in South Africa for both maternal and child. Having multiple children was found to be linked to poorer maternal well-being. There remains a need to improve social and psychological support among this population. Additionally, among adolescent mothers, delaying a second pregnancy until adulthood has benefits in terms of sibling effects relating to child cognitive development. Much of the existing literature on adolescent pregnancy focuses on prevention; however, sexual, reproductive health and family planning services may be required among adolescent mothers to provide choices relating to repeat pregnancy, child spacing and timing, accessing community and family support and childcare options. Adolescent mothers may still require support beyond giving birth to their first child. Existing intervention for the promotion of child cognitive development and family planning may be of benefit. Repeat pregnancies occur with growing frequency and the needs of both mothers and their children should be considered. Given the high rates of repeat pregnancies among adolescent mothers living with HIV, such services should be specifically adapted to integrate child and maternal mental and developmental care. It is a limited strategy to focus on prevention alone, and a rethink must be urgently undertaken to understand and provide for this growing group.

## Data Availability

Data are available on reasonable request.
